# Supplementary material for: Manual Lymph Drainage for Post-COVID-19 Related Cough, Breathlessness, and Fatigue; Two Case Reports
Source: Healthcare (Basel). 2023 Dec 1;11(23):3085. doi: 10.3390/healthcare11233085 (PMC10706012; doi:10.3390/healthcare11233085)
Supplement: Supplementary file 1 [file healthcare-11-03085-s001.zip › healthcare-2708077-supplementary.pdf]

INFORMED CONSENT FOR PUBLICATION OF CASE STUDY REPORT

Purpose of Case Study

The purpose of this case study is to explore the effect of Dr Vodder's Manual Lymph Drainage (MLD) on symptoms of Long COVID.

Long COVID as described on Wikipedia https://en.wikipedia.org/wiki/Long_COVID

Post COVID-19 condition (long-COVID) occurs in individuals with a history of SARS CoV-2 infection, usually 4 to 12 weeks from the onset of acute disease. Symptoms persist over time and cannot be explained by an alternative diagnosis, may persist from the initial illness, or have a new onset following and initial period of recovery and may fluctuate or relapse over time. Common symptoms can include fatigue, shortness of breath (asthmatic symptoms), cognitive dysfunction (brain fog) and joint/muscle pain, which have an adverse impact on everyday functioning. Symptoms and current approaches include providing persons affected with information on treating, recovery and managing symptoms.

Healthdirect.gov.au https://www.healthdirect.gov.au/covid-19/post-covid-symptoms-long-covid

Case Study Design

Participants will receive MLD treatments of up to one hour in duration. Treatments will be one week apart or upon availability. Treatments will be appropriate to the client and their presenting symptoms and will be performed by a therapist trained in the Dr Vodder Method of Manual Lymph Drainage.

The therapist will explain the proposed treatment, including any possible adverse reactions or events that could occur as a result of the treatment, to the participant before commencing the treatment.

The participant will be given the opportunity to ask questions regarding the treatment before giving consent to the treatment.

The therapist will confirm that the participant understands all aspects of the treatment before they give their consent to the treatment.

The therapist will explain the purpose of photographs and/or measurements being used (if applicable) and will explain the ways in which photographs and/or measurements may be used.

Participants will be required to complete a short questionnaire before and after each treatment and a follow up questionnaire 3 months after the first treatment.

Participants will be required to give their consent to receiving the treatments and to the use of the details resulting from the treatment which may include questionnaire answers, clinical records (including treatment notes) measurements and photos. All information included in the case study report will be de-identified to protect participant anonymity.

Participants will be given a copy of all documentation they sign giving consent.

I,_________________________ agree to take part in the case study, **The effects of Dr Vodder's Manual Lymph Drainage on Long COVID symptoms breathlessness and fatigue /_____________________ RMT/ MLD Therapist** which has been explained to me by __________________. I have read the participant information and any questions I had, have been answered.

Case Study Activities

I understand that

1. I may be interviewed by _________________________ **RMT/MLD Therapist** for details of my health history
2. My clinical records may be accessed and relevant details included in the case study publication
3. That Manual Lymphatic Drainage Techniques will be performed on presented symptoms as per case study.

4. I may be required to complete questionnaires before and after each treatment and a follow up questionnaire three months after the first treatment.
5. I may have measurements taken by _______________**RMT/MLD** Therapist at each treatment
6. I may have photographs taken by __________________**RMT/MLD Therapist** at each treatment

Voluntary Participation

I understand that

1. My participation is voluntary and that I can withdraw my information from the case report at any time without explanation and that this will not affect my treatment program or access to future treatment and information.

Privacy, Personal Information and Records Storage

I understand that

1. Only clinical data will be used in the case study report and that the resulting publication will not contain my name or personal information about me which has not been de-identified. I understand that there remains a possibility that someone may recognise me from reading the case study report. However, I understand that any information I provide is confidential, and that no information about me will be disclosed in any case study report, or to any other party, without first being de-identified.
2. Any [records of my participation including this consent form / interviews /clinical records] will be kept in secure storage [locked filing cabinet / password protected laptop / encrypted digital device / institution archive] and will be accessible to [therapist / researcher / institution staff] only.

A copy of this form has been provided to the participant Yes / No (please circle)

Signed

Participant: Full name: ____

Signature: ____

Date: ____

Or on behalf of participant:

Full name: ____

Signature: ____

Date: ____

Relationship to participant: __________________________________

Witness: Full name:____

Signature: ____

Date: ____

Position (in relation to the case study) RMT/MLD Therapist________
